# Unusual severe case of hemolytic uremic syndrome due to Shiga toxin 2d-producing *E. coli* O80:H2

**DOI:** 10.1007/s00467-017-3642-3

**Published:** 2017-03-25

**Authors:** Kioa L. Wijnsma, Anne M. Schijvens, John W. A. Rossen, A. M. D. (Mirjam) Kooistra-Smid, Michiel F. Schreuder, Nicole C. A. J. van de Kar

**Affiliations:** 1 Department of Paediatric Nephrology, Radboud University Medical Center, Amalia Children’s Hospital, PO Box 9101, 6500 HB Nijmegen, The Netherlands; 2 Department of Medical Microbiology, University of Groningen, University Medical Center Groningen, Hanzeplein 1, 9713 GZ Groningen, The Netherlands; 3 Department of Medical Microbiology, Certe Laboratory for Infectious Diseases, PO Box 909, 9700 AX Groningen, The Netherlands

**Keywords:** Hemolytic uremic syndrome, STEC-HUS, Serotype O80:H2

## Abstract

**Background:**

Hemolytic uremic syndrome (HUS) is one of the most common causes of acute renal failure in children, with the majority of cases caused by an infection with Shiga toxin-producing *Escherichia coli* (STEC). Whereas O157 is still the predominant STEC serotype, non-O157 serotypes are increasingly associated with STEC-HUS. However, little is known about this emerging and highly diverse group of non-O157 serotypes. With supportive therapy, STEC-HUS is often self-limiting, with occurrence of chronic sequelae in just a small proportion of patients.

**Case diagnosis/treatment:**

In this case report, we describe a 16-month-old boy with a highly severe and atypical presentation of STEC-HUS. Despite the presentation with multi-organ failure and extensive involvement of central nervous system due to extensive thrombotic microangiopathy (suggestive of atypical HUS), fecal diagnostics revealed an infection with the rare serotype: shiga toxin 2d-producing STEC O80:H2.

**Conclusions:**

This report underlines the importance of STEC diagnostic tests in all children with HUS, including those with an atypical presentation, and emphasizes the importance of molecular and serotyping assays to estimate the virulence of an STEC strain.

## Introduction

Hemolytic uremic syndrome (HUS) is the most common cause of acute renal failure in children and is diagnosed when the features of hemolytic anemia and thrombocytopenia are present simultaneously [1]. In over 90% of pediatric cases, HUS follows a gastro-intestinal infection with Shiga toxin-producing *Escherichia coli* (STEC), previously known by the name of post-diarrheal or typical HUS, because of its main characteristic of (bloody) diarrhea [1]. Whereas O157 is still the predominant STEC serotype, non-O157 serotypes are increasingly associated with STEC-HUS [2]. However, in contrast to STEC O157, little is known about this emerging and highly diverse group of non-O157 serotypes. Thrombotic microangiopathy (TMA) causing HUS predominantly affects the renal vasculature. Nevertheless, especially in non-O157 STEC serotypes, extra renal involvement such as central nervous system involvement is described [3]. If symptomatic treatment is started in time, the recovery of STEC-HUS is often spontaneously with minimal chronic sequelae [4].

Here we present a case of a 16-month-old boy with an unusual presentation and rare STEC serotype resulting in a very severe course of STEC-HUS.

## Case report

A previously healthy 16-month-old boy presented at the emergency department of a general hospital with convulsions and impaired consciousness. He had a 2-day history of coughing, vomiting, and lethargy, without signs of fever. Three months prior, he had a period of non-bloody diarrhea for over a week after visiting a biological farmer. A detailed medical history of the patient and family revealed no ingestion of unpasteurized milk or cheese nor a visit to a foreign country. On physical examination he exhibited fluctuating levels of alertness. His respiratory rate was 29/min with oxygen saturation of 97%. Body temperature (36.4 °C) and blood pressure were normal (98/60 mmHg) with mildly elevated pulse rate (138 beats per minute). Examination of the heart, lungs, and abdomen was unremarkable. Skin examination revealed a pale-looking patient with petechiae at lower limbs.

Based on clinical suspicion of meningitis or non-convulsive status epilepticus, ceftriaxone and midazolam were administered and the patient was transferred to our hospital. Initial laboratory evaluation showed hyperglycemia and the triad of HUS (Table 1). In the first hours, consciousness further decreased and the patient was transferred to the pediatric intensive care unit (ICU), where peritoneal dialysis was initiated due to persistent anuria. Blood and feces samples to determine the presence of an STEC infection were obtained. Blood cultures were performed repeatedly and remained negative during admission. Thrombotic thrombocytopenic purpura (TTP) was excluded with a normal ADAMTS13 activity (Table 1). Atypical HUS (aHUS), caused by complement dysregulation, was considered because of severity of the presentation, mainly neurological presentation, no recent history of (bloody) diarrhea, and young age of the patient. Consequently, the patient received 600 mg of eculizumab. Later incoming results of additional complement and DNA diagnostics showed no abnormalities (Table 1).

**Table 1:** Multi-organ involvement

	Test	At admission	Most abnormal value	At discharge	Normal value or range
Differential diagnosis					
	Blood culture	Negative		Negative	
*TTP*	ADAMTS13 activity (%)	91%			>65%
*STEC-HUS*	Serology				
	O157 IgM/G/A	Negative			
	O26 IgM/G/A	Negative			
	PCR *Stx1 gene*	Negative		Negative	
	*Stx2 gene*	Positive		Negative	
	*eae gene*	Positive		Negative	
	WGS Serotype	O80:H2			
	Isolate	Stx2d, *eae* ξ variant			
	Sequence type	ST301			
	Virulence factors				
	*Stx1gene*	Negative			
	*Stx2gene*	Positive			
	*eae*	Positive			
	*aggR*	Negative			
	*aatA*	Negative			
*aHUS*	Complement				
	C3 (mg/l)	874	NA	NA	900–1800
	C4 (mg/l)	71	NA	NA	150–400
	Anti factor H autoantibodies	Negative			
	DNA analysis				
	*Factor H*	No pathogenic variation			
	*Factor I*	No pathogenic variation			
	*Factor B*	No pathogenic variation			
	*C3*	No pathogenic variation			
	*MCP*	No pathogenic variation			
	*CFHR 1–5*	No pathogenic variation			
	*DGKE*	No pathogenic variation			
	*THBD*	No pathogenic variation			
	*MLPA* *Factor H operon*	No aberrations			
Hematological	Hemoglobin (mmol/l)	4.5	3.3	7.3	6.8–8.6
	Platelet count (x10^9^/l)	22	18	872	210–430
	WBC count (x10^9^/l)	15.5	29.0	12.9	5.0–17.0
	Schizocytes (%)	>5	>5	NA	<0,5
	Haptoglobin (g/l)	Hemolytic	Hemolytic	1.85^a^	0.3–1.6
	LDH (U/l)	2285	6521	908	<250
Kidney	Creatinine (μmol/l)	167	444 (PD)	470 (PD)	15–45
	eGFR(ml/min/1.73 m^2^)	17	anuria	anuria	80–120
	BUN (mmol/l)	43.9	44.2	22.4	2.5–7.0
Brain	MRI	Diffusion restriction of the deep white matter consistent with metabolic encephalopathy	NA	NA	
	EEG	No epileptic activity	NA	NA	
Heart	CK (U/l)	2743	8390	139^a^	<170
	Ntpro-BNP (pg/ml)	NA	>180,000	NA	<320
	Troponin T levels (ng/l)	557	23,444	508^a^	<14
	Echo	NA	Left ventricular dysfunction	Normal left ventricular function^a^	
Pancreas	Amylase (U/l)	NA	1933	42^a^	<105
	Glucose (mmol/l)	9.9	30.7	6.2	4.0–5.6
	Triglycerides (mmol/l)	NA	14.17	4.92	0.8–2.0
Liver	AST (U/l)	163	1020	114	<35
	ALT (U/l)	62	480	144	<45
	Gamma-GT (U/l)	6	1810	824	<55
	Alkaline phosphatase (U/l)	205	1529	401	<115
	Direct bilirubin (μmol/l)	4	326	35	<5
	Ultrasound		Edema around and sludge inside gallbladder		
	Biopsy		Cholestasis		

*aatA* necessary for translocation of dispersin (Aap), *ADAMTS13* a disintegrin and metalloproteinase with a thrombospondin type 1 motif, member 13, *aggR* transcriptional regulator aggR, *aHUS* atypical hemolytic uremic syndrome, *ALT* alanine transaminase, *AST* aspartate transaminase, *BUN* blood urea nitrogen, *CFHR* complement factor H-related proteins, *CK* creatine kinase, *DGKE* diacylglycerol kinase epsilon, *eae E. coli* attachment effacement gene (intimin), *EEG* electroencephalogram, *eGFR* estimated glomerular filtration rate based on Schwartz estimation with k-value of 36.5, *gammaGT* gamma-glutamyl transferase, *IgA* immunoglobulin A, *IgG* immunoglobulin G, *IgM* immunoglobulin M, *LDH* lactate dehydrogenase, *MCP* membrane cofactor protein, *MLPA* multiplex ligation-dependent probe amplification, *MRI* magnetic resonance imaging, *NA* not available, *Ntpro-BNP* N-terminal of the prohormone brain natriuretic peptide, *PCR* polymerase chain reaction, *PD* peritoneal dialysis, *STEC* Shiga toxin-producing *Escherichia coli, Stx1* Shiga toxin 1, *Stx2* Shiga toxin 2, *THBD* thrombomodulin, *TTP* thrombotic thrombocytopenic purpura, *WBC* white blood cell count, *WGS* whole genome sequencing

^a^Two months after presentation

The following day, fecal diagnostics revealed an STEC infection, indicating STEC-HUS; real-time polymerase chain reaction (PCR) was positive for Shiga toxin 2 (*Stx2*) and attaching and effacing (*eae*) genes. In addition, molecular serotyping using whole genome sequencing revealed the rare STEC serotype O80:H2, which contained the *Stx*2d gene and the rarely in human seen eae ξ gene variant (Table 1) [5].

In the course of admission, the patient developed multiple signs of severe, extra renal manifestations of TMA. Due to the severe neurological symptoms, with convulsions and decreased consciousness, brain magnetic resonance imaging (MRI) was performed, showing diffusion restriction of the deep white matter (Table 1).

On day four of admission, the patient was resuscitated twice due to post-intubation hypotension and bradycardia, with rapid recovery of cardiac output. Cardiac biomarkers were elevated, and retrospectively elevated troponin T levels and creatine kinase were already present. Echocardiogram revealed a mildly dilated and dysfunctional left ventricle. Eventually, a follow-up demonstrated a significant improvement in cardiac biomarkers and function.

Pancreas involvement was noticed based on a gradual rise in serum glucose and elevation of serum amylase and triglyceride concentrations without clinical signs of pancreatitis. Insulin treatment was needed for 2 weeks.

**Fig. 1:**
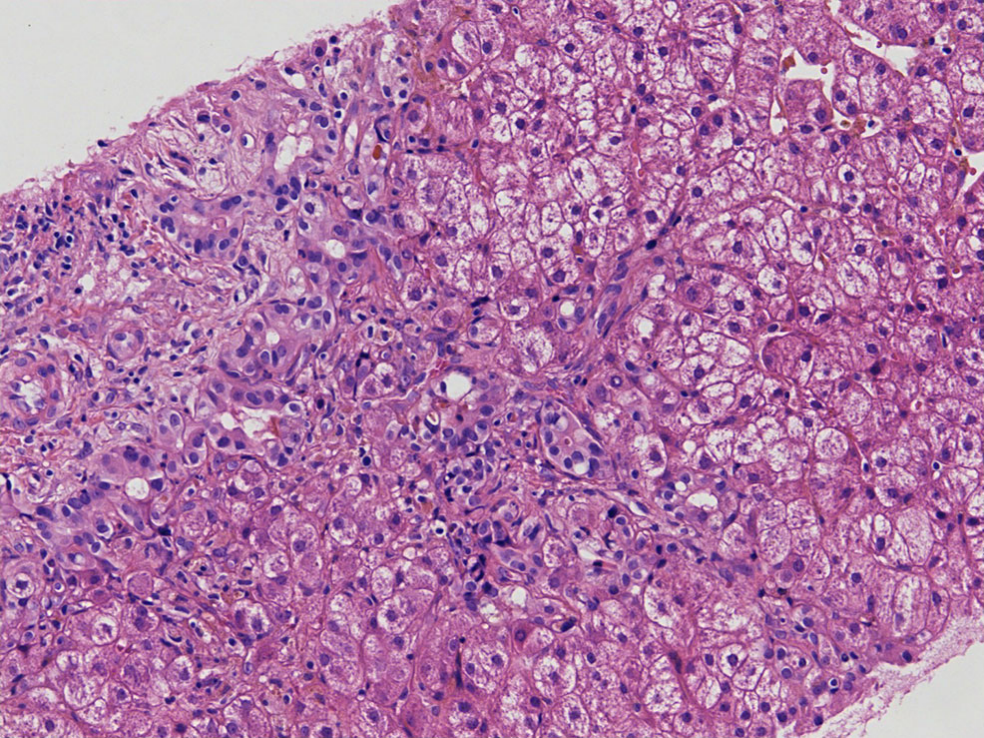
Light microscopy on the liver biopsy (with hematoxylin and eosin staining) revealed hepatocyte swelling and extensive signs of cholestasis

After 2 weeks, jaundice was observed in combination with elevated bilirubin levels, without signs of ongoing hemolysis. Progressive elevation of transaminases was measured, with normal levels of clotting factors but low serum albumin (Table 1). Liver biopsy showed extensive signs of cholestasis without microthrombi, most likely due to sludging or drug-related effects (Fig. 1).

Eventually, our patient was hospitalized for over 3 months. Only minimal signs of neurological improvement were observed and over time the patient developed spastic dystonia. Furthermore, he exhibited persistently decreased levels of consciousness with frequent periods of severe agitation. The patient remained anuric for which dialysis was continued. Nine months after initial presentation, the patient died due to peritoneal and hemodialysis failure combined with progressive liver failure and further neurological impairment.

## Discussion

In this report, we describe a 16-month-old boy with an unusual, severe presentation and course of STEC-HUS due to the rare *Stx*2d-producing *E. coli* O80:H2. STEC-HUS is a common cause of HUS in childhood and in more than half of the cases results from an STEC infection with serotype O157. However, non-O157 serotypes are increasingly associated with HUS in recent years, most likely due to improvement of diagnostics [1, 2]. Usually, the majority of STEC-HUS resolves with no or minimal chronic sequelae.

STEC infection with serotype O80:H2 resulting in HUS was rarely described up to recently [2, 6–8]. To our knowledge, up till now, this serotype was only found in patients with HUS in France and this report is the first describing a case in the Netherlands. In addition, one study from Spain already described the finding of this serotype in cattle over 10 years ago. Interesting to note is that there is no direct geographic connection between the different areas. Hence, the source of the O80 serotype remains of unknown origin [8].

STEC strains can produce different shiga toxins, respectively *Stx1* and *Stx2*. Within these genotypes, especially the *Stx2 gene*, there is a wide variety of *Stx* subtypes, such as *Stx2d* which we describe in this Case report [9]. Moreover, the *Stx2*d gene is known to cause a more severe course of disease. This together with the rare *eae* ξ and the plasmid of the O80:H2 STEC, which showed high resemblance with the previous described plasmid associated with extra intestinal virulence, resulted in an increased association with HUS [8]. Only recently, Soysal et al. described the STEC serotype O80 as a new therapeutic challenge in patients with STEC-HUS due to its capacity to cause a bacteremia [8]. Furthermore, Espié et al. described an outbreak of STEC serotypes O26 and O80 co-infection related to unpasteurized cheese [6]. However, our patient had no bacteremia and no indication of co-infection, tested with both fecal diagnostics and serological antibody assays against both O157 and O26 lipopolysaccharide (Table 1).

Mariani-Kurkdjian et al. described the case of an adult male with a severe episode of HUS caused by STEC O80:H2 containing both *Stx*2d and the rare *eae* ξ variant like the strain described above [7]. Similar to our case, the adult male patient presented with afebrile convulsions followed by coma and, after a few weeks, he developed bacteremia. This raised the question if the O80 serotype may not be cleared rapidly, in contrast to most other serotypes. Such a slow clearance would make it more likely that the STEC found in our patient could be the consequence of the gastro-intestinal infection three months prior to presentation [8, 10].

Nowadays, in the majority of laboratories, STEC diagnostics comprises PCR, culture, and/or enzyme immune-assays. Obviously, these approaches could have missed the identification of the O80:H2 strain. Using additional molecular and serotyping assays allows the detection of virulence factors and typing of the *Stx* genes, both important to estimate the virulence of the strain. Such additional diagnostic tests are relevant both for individual patients as for public health to monitor new or rare serotypes that cause severe HUS and emerge into the population [9].

It can be challenging to clinically differentiate between STEC-HUS and aHUS due to similarity of symptoms. Similar to our patient, in 6–10% of the children with STEC-HUS there is no (bloody) diarrhea, whereas aHUS is preceded by diarrhea in 25% of cases [11]. Since aHUS is merely a diagnosis *per exclusionem*, it is essential to prove the absence of an STEC infection [1, 12]. Even in cases without (bloody) diarrhea and atypical presentations such as young age of the patient, it is highly recommended to perform both fecal and serological diagnostics in every HUS patient to exclude STEC-HUS [12].

The treatment of STEC-HUS is merely symptomatic. A topic of discussion remains the use of antibiotics to eradicate STEC infection. Increased transcription, production, and release of shiga toxins, possibly aggravating HUS, can be induced by antibiotics [13]. Soysal et al. studied in vitro effects of different antibiotics on *Stx* production. Ceftriaxone, which our patient received at presentation, seems to have no effect on *Stx* production [8]. Another controversial topic in patients with STEC-HUS is the use of eculizumab. Eculizumab, a monoclonal antibody directed against complement C5, is nowadays the standard treatment in patients with aHUS [14]. Some case series, among others Pape et al., reported better outcome of STEC-HUS after eculizumab administration, particularly in neurologically affected STEC-HUS patients [3, 15]. However, Pape et al. also described the use of eculizumab in patients with multi-organ failure, where it seems to be associated with a less favorable outcome [15]. Well-designed and well-powered randomized controlled trials are needed to shed light on the effect of eculizumab in STEC-HUS.

Extra-renal manifestations of TMA are not uncommon in HUS. In 25% of HUS cases neurological involvement is noted, other organs are affected less frequently [1, 3]. Although severe multi-organ failure and especially liver failure is rarely described in STEC-HUS, the highly severe disease presentation in our patient could partially be explained by the (extraintestinal) virulence factors of this O80 strain. However, in our patient, jaundice and liver failure evolved 2 weeks after one dose of eculizumab possibly indicating hepatotoxicity. This was recently described in five pediatric aHUS patients [16]. Transient liver enzyme derangement was noticed in these children 10–29 days after the first dose of eculizumab with spontaneous resolution. The time pattern in our patient, as described above, cannot rule out the hypothesis that administration of eculizumab could have contributed to liver failure in our patient.

In conclusion, we describe a severe case of STEC-HUS caused by an unusual *Stx*2d-producing STEC O80:H2. This resulted in an unusual and severe disease course, complicated by multi-organ failure and central nervous system involvement. This case emphasizes the importance of molecular and serotyping assays to estimate the virulence of an STEC strain.
